# Adherence to Breast, Cervical, and Colorectal Cancer Screening Among Women in Spain: Evidence from the 2023 Spanish Health Survey

**DOI:** 10.3390/healthcare14142175

**Published:** 2026-07-18

**Authors:** Lola Ibañez-Gonzalez, Rodrigo Jiménez-García, José J. Zamorano-Leon, Ana Jimenez-Sierra, Lucia Fuentes-Arroyo, David Carabantes-Alarcon, Ana López-de-Andrés, María Teresa Saez-Vaquero, Carlos Llamas-Saez

**Affiliations:** 1 Department of Public Health and Maternal & Child Health, Faculty of Medicine, Universidad Complutense de Madrid, 28040 Madrid, Spain; lolaiban@ucm.es (L.I.-G.); rodrijim@ucm.es (R.J.-G.); luciaifu@ucm.es (L.F.-A.); dcaraban@ucm.es (D.C.-A.); carlolla@ucm.es (C.L.-S.); 2 Instituto de Investigación Sanitaria del Hospital Clínico San Carlos (IdISSC), 28040 Madrid, Spain; anailo04@ucm.es; 3 Faculty of Medicine, Universidad CEU San Pablo, 28003 Madrid, Spain; a.jimenez100@usp.ceu.es; 4 Department of Public Health and Maternal & Child Health, Faculty of Pharmacy, Universidad Complutense de Madrid, 28040 Madrid, Spain; 5 Servicio Madrileño de Salud, Consejeria De Sanidad, 28046 Madrid, Spain; mteresa.saezva@salud.madrid.org

**Keywords:** cancer screening, mammography, cervical cytology, colorectal cancer screening, fecal occult blood test, social support

## Abstract

**Background/Objectives**: Cancer screening is a key strategy for reducing cancer-related morbidity and mortality. This study assessed adherence to breast, cervical, and colorectal cancer screening among Spanish women and identified factors independently associated with participation. **Methods**: A cross-sectional study was conducted using data from the 2023 Spanish Health Survey. Women eligible for mammography (40–69 years), cervical cytology (25–65 years), and fecal occult blood testing (FOBT; 50–69 years) were included. Weighted prevalence estimates were calculated, and multivariable logistic regression models were fitted separately for each screening test. **Results**: A total of 7454 women aged 25–69 years were analyzed. Adherence was highest for mammography (71.1%), followed by cervical cytology (64.1%) and FOBT (36.8%). Only 24.4% of women eligible for all three programs were adherent to all screening tests simultaneously. Across all screening programs, adherence was consistently associated with being born in Spain, living with a partner, greater social support, healthcare utilization, private health insurance coverage, and physical activity. Strong social support was independently associated with participation in mammography (OR 1.72; 95% CI 1.35–2.20), cervical cytology (OR 1.58; 95% CI 1.29–1.94), and FOBT uptake. Participation in one screening program was strongly associated with participation in the others, particularly between mammography and cervical cytology (OR 4.37; 95% CI 3.84–4.98). **Conclusions**: Adherence to cancer screening among Spanish women remains suboptimal, particularly for colorectal cancer. Participation appears to reflect a broader preventive health behavior pattern, with social support emerging as one of the strongest and most consistent determinants across all three screening programs.

## 1. Introduction

Cancer represents a substantial global burden among women, with persistent inequalities in early detection, treatment, and survival both between and within countries [1,2]. Among women, breast cancer is the most frequently diagnosed malignancy and the leading cause of cancer-related death, while colorectal and cervical cancers continue to contribute substantially to morbidity and mortality worldwide [3,4,5].

In Spain, according to the National Statistics Institute (Instituto Nacional de Estadística), cancer accounts for 22% of all deaths among women and is the second leading cause of mortality after cardiovascular diseases [6]. Breast cancer is the most common malignancy, with approximately 34,000 new cases diagnosed annually, and represents a major cause of mortality, with a mortality rate of 21.2 deaths per 100,000 women [7]. Colorectal cancer also represents a considerable disease burden, with approximately 18,000–19,000 new diagnoses annually and a mortality rate comparable to that observed for breast cancer [7]. Cervical cancer, in contrast, has a substantially lower incidence, with approximately 2200–2300 new cases and around 700 deaths annually.

Cancer accounts for approximately 4–6% of total healthcare expenditure in Spain, representing several billion euros annually in direct healthcare costs [8].

In this context, cancer screening constitutes a cornerstone of public health strategies. Europe’s Beating Cancer Plan places early detection at the center of cancer control policies [9]. At the European level, there is broad consensus regarding the recommended minimal recommendation of the following screening programs: biennial mammography screening for breast cancer in women aged 50–69 years; biennial fecal occult blood testing (FOBT) for colorectal cancer in both sexes aged 50–69 years; and population-based cervical cancer screening through cervical cytology every 3 years in women aged 25–35 years, together with primary human papillomavirus testing every 5 years in women aged ≥35 years and never before the age of 30 years [10].

For breast cancer screening, European guidelines establish that participation rates of at least 70% represent the minimum threshold required to achieve a significant reduction in mortality, while participation levels ≥ 75% are considered desirable to maximize population-level benefits [11]. In colorectal cancer screening, participation rates of approximately 45–50% are considered sufficient to achieve measurable reductions in mortality, although levels ≥ 65–70% clearly optimize program effectiveness [12]. Regarding cervical cancer screening, participation rates close to 70% are associated with substantial reductions in incidence and mortality, whereas more recent European strategies have proposed targets exceeding 90% to advance toward the elimination of this preventable malignancy [13].

The Spanish National Health System Cancer Strategy is aligned with European Union recommendations and includes these three screening programs within its portfolio of healthcare services, although with varying degrees of implementation, participation, and effective coverage [14]. Breast cancer screening, introduced in 1990, currently covers the entire target population, with participation rates approaching 74%, slightly above the European Union average, estimated at approximately 65–70% according to international comparative data [8,15]. Cervical cancer screening, which transitioned from an opportunistic to a population-based model beginning in 2019 [16], shows participation rates among eligible women of around 68%, in line with average European levels (approximately 60–65%) [8,15]. Colorectal cancer screening, incorporated into the healthcare system in 2014 and progressively implemented across the autonomous communities, has participation rates close to 32%. Despite improvements observed in recent years, these rates remain below both European averages (approximately 40–50%) and recommended targets, with substantial regional variability [8,15]. Regarding temporal trends, participation in breast and cervical cancer screening programs has remained relatively stable over the last decade, whereas colorectal cancer screening has shown an upward trend, although still insufficient to meet established objectives [8,15].

International recommendations consistently emphasize that identifying barriers and determinants of participation is essential for the success of cancer screening programs, as their effectiveness depends not only on the availability of interventions but also on equitable access and population adherence [9,16]. The World Health Organization and the International Agency for Research on Cancer underline that participation in cancer screening follows a multifactorial model involving structural, healthcare system–related, and individual determinants, whose interaction influences both health outcomes and associated inequalities [5,17]. Previous studies conducted in Spain and elsewhere have highlighted the importance of sociodemographic variables such as income, educational level, nationality, social support, use of and access to health services, and lifestyles, among other factors [18,19,20,21,22,23,24,25,26,27,28,29,30]. Although these studies have examined determinants of participation in individual cancer screening programs, few have simultaneously evaluated adherence to breast, cervical, and colorectal cancer screening within the same nationally representative population. Moreover, the role of social support and the extent to which participation in one screening program is associated with adherence to other cancer screening tests remain insufficiently explored [18,19,20,21,22,23,24,25,26,27,28,29,30]. Understanding whether common determinants influence participation across different screening programs may help identify population groups at risk of underutilization of preventive services and support the development of integrated screening strategies.

The aims of this study were to describe adherence to breast, cervical, and colorectal cancer screening programs among Spanish women according to sociodemographic, healthcare utilization, and clinical variables, using data from the 2023 Spanish National Health Survey, and to identify, using multivariable analysis, which of these variables were independently associated with adherence to each of the three cancer screening programs evaluated.

## 2. Materials and Methods

### 2.1. Study Design and Data Source

We conducted a cross-sectional study using data from the 2023 Spanish Health Survey (Encuesta de Salud de España, ESdE 2023) [31]. The ESdE 2023 is a nationally representative health survey conducted by the Spanish Ministry of Health and the National Statistics Institute (INE) [1]. ESdE 2023 uses a stratified three-stage sampling design to obtain nationally and regionally representative estimates of the non-institutionalized population living in Spain. The first stage units are the census tracts. The second-stage units are the main family homes. Within each household, an adult (15 years or older) is randomly selected (third stage) to complete the adult questionnaire. The ESdE 2023 was designed to provide representative estimates at regional (Autonomous communities) and national levels [31].

Data was collected between September 2023 and August 2024, using a mixed Computer-Assisted Personal Interviewing (CAPI) and Computer-Assisted Web Interviewing (CAWI) data collection system. Information from 21.085 households was collected in the final sample, and 21,040 adults (15 years or older) were interviewed [31].

### 2.2. Participants

The study population included women who completed the adult questionnaire of the ESdE 2023. For each of the three cancer screening tests evaluated in this investigation (mammography, cervical cytology, and FOBT), we selected women within the actual recommended age groups. So, we included women aged 40–69 years for breast cancer screening, 25–65 years for cervical cancer screening, and 50–69 years for colorectal cancer screening [14]. The recommended age for mammography used in our study was reduced to 40 years, as this is the recommended age for women with a family history of breast cancer by public health authorities and in the general population by Spanish scientific societies and private healthcare systems, and has been previously used in other Spanish studies [14,20,32]. Therefore, we have used a survey-based operational definition of recent screening for mammography, not necessarily strict program adherence according to all guideline intervals [14,20,32].

### 2.3. Variables

The outcome variables were the adherence to mammography, cervical cytology, and FOBT. Adherence was defined according to self-reported participation and time since the last mammography, cervical cytology, or FOBT. Detailed question wording, definitions, and categorization criteria are shown in Supplementary Table S1.

Adherence to mammography and cervical cytology uptake was defined as having undergone the test within the previous 36 months, and FOBT within the previous 24 months. These time frames were decided to make the results comparable to other previous studies conducted in Spain with population surveys and to reduce possible underestimation resulting from inaccurate recall or minor delays in care [18,20,21,22,33,34].

Independent variables were all self-reported and grouped into four domains: (a) sociodemographic characteristics, (b) healthcare services use, (c) clinical characteristics, and (d) lifestyle factors. Sociodemographic characteristics included age, country of birth, living with a partner, educational level, employment status, monthly household income, social support, and caregiver status.

Social support was assessed using the Oslo Social Support Scale, a validated instrument widely used in population-based studies [35].

Healthcare utilization services variables included: primary care doctor visit in the previous 12 months, specialist visit in the previous 12 months, emergency room visit in the previous 12 months, and private health insurance coverage.

Clinical characteristics collected were: self-rated health, any chronic condition, and grouped chronic disease categories (heart disease and stroke, musculoskeletal disease, respiratory disease, chronic gastrointestinal disorder, neurological disorder, mental health disorders, and cancer). Lifestyle variables included alcohol use, tobacco use, free time physical activity, and body mass index. The question’s wording and categorizing criteria for all study variables are provided in Table S1.

### 2.4. Statistical Analysis

All analyses incorporated the sampling weights provided by the ESdE 2023 and accounted for the complex survey design using Stata survey procedures, thereby generating nationally representative estimates.

Absolute frequencies and percentages were calculated to describe the characteristics of the entire study population (women aged 25–69 years) and the adherence to mammography, cervical cytology, and FOBT according to the independent variables.

The bivariate association of study variables with adherence to each of the three screening tests was assessed using the Chi-square test or Fisher’s exact test as appropriate. However, because multiple bivariate comparisons were performed, these analyses were considered exploratory and descriptive. The primary inferences of the study are based on the results of multivariable analysis; therefore, isolated statistically significant findings from the bivariate analyses should be interpreted with caution.

Multivariable logistic regression models were fitted separately for mammography, cervical cytology, and FOBT adherence to identify variables independently associated with each screening test. Variables associated with the adherence in the bivariate analysis (*p* < 0.20) and those considered clinically or scientifically relevant based on previous literature were entered into the initial multivariable logistic regression model. Model building followed a stepwise approach [36]. At each step, the contribution of individual variables was evaluated using Wald statistics, and changes in the estimated regression coefficients were examined by comparing the multivariable estimates with those obtained from the corresponding univariable models to identify potential confounding. Variables that did not contribute meaningfully to the model and did not act as confounders were sequentially removed. After each elimination, the reduced model was compared with the preceding model using the Likelihood Ratio Test. This iterative process continued until a parsimonious final model was obtained. Before fitting the final models, multicollinearity among the independent variables was assessed using variance inflation factors (VIFs). No evidence of problematic multicollinearity was identified, and all retained variables met the predefined diagnostic criteria. The final model was assessed for potential two-way interactions among the retained variables. The measure of association obtained was the adjusted odds ratio (OR) with its 95% confidence interval (95% CI).

Finally, to assess the association between adherence to different screening tests, additional logistic regression models were fitted, estimating the odds of adherence to one screening test according to adherence to another screening test.

The statistical software used was Stata 15.1 (StataCorp LLC, College Station, TX, USA). A two-tailed *p*-value < 0.05 was considered statistically significant.

### 2.5. Sensitivity Analysis

Adherence to mammography analyses was repeated after restricting the study population to women aged 50–69 years, corresponding to the target age range of the organized national population-based breast cancer screening program in Spain. Baseline characteristics and mammography adherence were described according to all study variables, and an additional multivariable logistic regression model was fitted to identify factors independently associated with mammography uptake in this population.

### 2.6. Ethics

We used publicly available, anonymized secondary data from ESdE 2023. The database can be freely downloaded from the Spanish Ministry of Health [37]. Because this is a descriptive observational study based on anonymized secondary data, according to the Spanish legislation, ethical approval and informed consent were waived [38]. No intervention was carried out on the participants. This study was conducted in accordance with the ethical principles of the Declaration of Helsinki and complied with Regulation (European Union) 2016/679 (General Data Protection Regulation) and Organic Law 3/2018 on Personal Data Protection and Digital Rights [39,40].

## 3. Results

**Figure 1:**
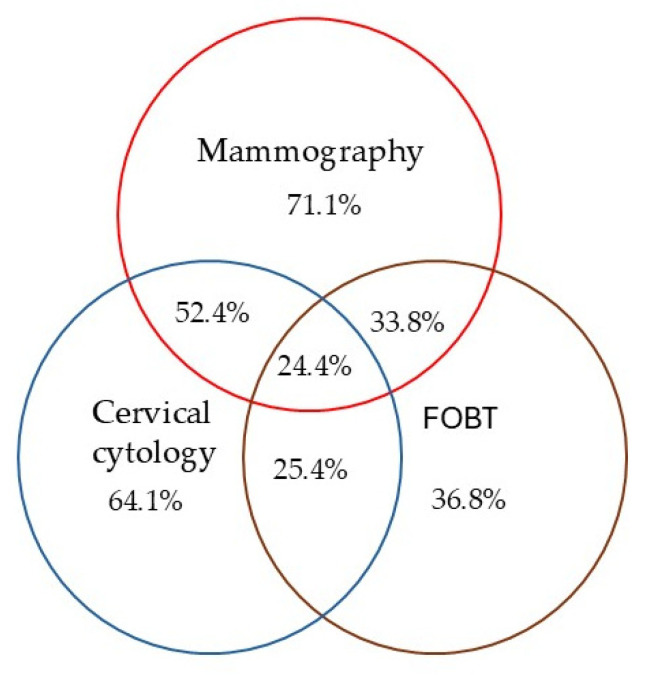
Adherence to mammography, cervical cytology and fecal occult blood (FOBT) test among women interviewed in the Spanish National Health Survey 2023.

A total of 7454 women aged 25–69 years were included in the study (Table S2). The age distribution was balanced across age groups. Most participants were born in Spain (82.9%), lived with a partner (53.8%), and were employed (61.4%). Approximately one-third had completed university studies (32.0%), while 25.1% reported household monthly incomes below €1650. Most women reported moderate or strong social support (93.3%), and 14.0% identified themselves as caregivers. Regarding healthcare utilization, 83.5% had visited a primary care physician, and 64.6% had seen a specialist physician during the previous 12 months. Overall, 62.7% reported at least one chronic condition, musculoskeletal diseases being the most frequent (32.3%). Additionally, 20.9% of women were current smokers, 27.3% reported daily or weekly alcohol consumption, and 27.1% engaged in regular leisure-time physical activity (Table S2). Figure 1 shows substantial differences in adherence across the three cancer screening tests evaluated. Mammography showed the highest adherence, with 71.1% of women within the recommended target group reporting uptake within the established interval, followed by cervical cytology (64.1%). In contrast, adherence to FOBT was markedly lower (36.8%). Regarding combined screening uptake, 52.4% of women eligible for both mammography and cervical cytology adhered to both screening tests simultaneously. Lower proportions were observed for combinations involving FOBT, with 33.8% of women adherent to both mammography and FOBT, and 25.4% adherent to both cervical cytology and FOBT. Notably, fewer than one in four women eligible for all three screening programs were adherent to mammography, cervical cytology, and FOBT simultaneously (Figure 1).

Adherence to mammography increased markedly with age, reaching the highest prevalence among women aged 52–57 years (84.8%) and remaining above 78% in older age groups (Table 1). Higher uptake was also observed among women born in Spain, those living with a partner, women reporting strong social support, and caregivers. Regarding healthcare-related variables, mammography adherence was significantly higher among women who had attended primary care or specialist consultations during the previous year and among those with private health insurance coverage (Table 2). Women with chronic conditions, particularly musculoskeletal diseases and cancer (86.0%), also showed higher participation in breast cancer screening.

In the multivariable analysis, older age was the factor most strongly associated with mammography adherence, with the highest odds observed among women aged 52–57 years (OR 5.49; 95% CI 4.41–6.82). Other variables associated with higher adherence included being born in Spain, living with a partner, having moderate or strong social support, visiting primary care or specialist physicians during the previous year, having private health insurance coverage, having musculoskeletal diseases, having a history of cancer, and being physically active. The strongest associations, apart from age, were observed for private health insurance coverage (OR 1.85; 95% CI 1.49–2.29), strong social support (OR 1.72; 95% CI 1.35–2.20), history of cancer (OR 1.70; 95% CI 1.20–2.41), and physical activity (OR 1.68; 95% CI 1.43–1.97) (Table 3).

The results of the sensitivity analysis restricted to women aged 50–69 years are shown in Tables S3 and S4. Mammography uptake was 81.0%, and the distribution of adherence according to sociodemographic, healthcare-related, clinical, and lifestyle characteristics closely resembled that observed in the main analysis. Likewise, the multivariable logistic regression identified essentially the same independent predictors of mammography adherence, with only minor differences in the magnitude of the estimated odds ratios (Tables S3 and S4).

Cervical cytology adherence was highest among women aged 35–54 years (over 67%) and decreased among older women, reaching 59.0% in those aged 55–65 years (Table 1). Participation was significantly greater among women born in Spain, those living with a partner, women with higher educational attainment, strong social support, and caregivers. Uptake was also higher among women who had visited healthcare services during the previous year, particularly specialist physicians, and among those with private health insurance coverage, whose adherence exceeded 80% (Table 4).

In the multivariable analysis, women aged 35–44 years were more likely to adhere to cervical cytology screening than those aged 25–34 years. Private health insurance coverage (OR 2.20; 95% CI 1.83–2.65), university education (OR 1.64; 95% CI 1.37–1.97), and specialist physician visits (OR 1.62; 95% CI 1.43–1.84) showed the strongest associations. Spanish-born status, living with a partner, moderate social support (OR 1.26; 95% CI 1.02–1.55), and strong social support (OR 1.58; 95% CI 1.29–1.94), musculoskeletal diseases, cancer, and physical activity were also associated with higher adherence. Conversely, overweight and obesity were independently associated with lower cervical cytology adherence (Table 5).

FOBT adherence was substantially lower than the adherence observed for mammography and cervical cytology. Participation increased with age, peaking among women aged 55–59 years (41.9%) (Table 1). Higher adherence was observed among women born in Spain, those living with a partner, women with higher educational attainment, those with strong social support, and caregivers. Women who had used healthcare services during the previous year showed significantly greater FOBT uptake, particularly those attending primary care consultations (39.4% vs. 18.7% among non-users). Higher participation was also observed among women with chronic conditions, poorer self-rated health, musculoskeletal and gastrointestinal diseases, cancer, and among physically active women (Table 6).

In the multivariable logistic regression analysis, older age, Spanish-born status, living with a partner, strong social support, higher educational attainment, recent primary care and specialist visits, private insurance coverage, musculoskeletal and gastrointestinal diseases, cancer, and physical activity remained independently associated with greater FOBT uptake. Recent primary care physician visits emerged as the variable most significantly associated with FOBT adherence (OR 2.10; 95% CI 1.61–2.74). In contrast, women reporting very good or good self-rated health showed lower odds of FOBT adherence (OR 0.84; 95% CI 0.72–0.97) (Table 7).

Finally, adherence to one cancer screening program was consistently associated with participation in other screening tests, suggesting the existence of a common preventive health behavior pattern among women (Table 8). The strongest association was observed between mammography and cervical cytology uptake, with women adherent to one of these tests showing more than fourfold higher odds of adherence to the other (OR 4.37; 95% CI 3.84–4.98). Similarly, women adherent to mammography were significantly more likely to participate in FOBT screening (OR 3.88; 95% CI 3.14–4.81). A weaker, although still statistically significant, association was found between cervical cytology and FOBT uptake (OR 1.69; 95% CI 1.45–1.97).

## 4. Discussion

Our findings suggest that participation in cancer screening among Spanish women reflects a broader preventive health behavior pattern rather than isolated decisions regarding individual screening tests. Adherence was highest for breast cancer screening, intermediate for cervical cancer screening, and lowest for colorectal cancer screening. Furthermore, participation in one screening program was strongly associated with participation in the others, suggesting the existence of a common profile of adherence to preventive healthcare recommendations. Across all three screening programs, greater healthcare utilization, complementary private health insurance coverage, physical activity, living with a partner, being born in Spain, and higher levels of social support were consistently associated with increased participation. Particularly noteworthy was the consistency of social support across all multivariable models, suggesting that it may represent a previously underrecognized cross-cutting determinant of preventive healthcare engagement.

The differences observed between screening programs appear to be largely explained by their distinct organizational trajectories [18]. The high participation observed in breast cancer screening is consistent with the maturity and consolidation of the Spanish breast cancer screening program, as previously described by Molina-Barceló et al. [18]. Using data from the 2017 Spanish National Health Survey, Álvarez-González et al. reported a participation rate of 66.8% among women aged 40–69 years, slightly lower than that observed in the present study [41]. Nevertheless, participation remains below that reported in well-established population-based screening programs such as those implemented in Denmark and the Netherlands [19,42].

Cervical cancer screening occupied an intermediate position, consistent with the ongoing transition in Spain from an opportunistic to a fully population-based screening model. Compared with previous national survey-based studies, we found no substantial improvements in adherence to cervical cancer screening over recent years [20,21], with current participation remaining close to the European average [43]. In contrast, countries such as Australia, where organized cervical cancer screening programs are well established, have reported substantially higher participation rates [44]. International studies have highlighted the potential of alternative organizational strategies, including active invitation systems and self-sampling approaches, to improve participation [45,46,47]. However, further research is needed to assess the feasibility and effectiveness of implementing such strategies at the national level in Spain [33].

The lower adherence observed for colorectal cancer screening is consistent with the most recent Spanish studies by Portero de la Cruz and Cebrino, and by Gisbert Canet et al., which reported participation rates close to 38.7% [22,34]. Although participation is higher in countries with more mature colorectal screening programs, such as the Netherlands and Australia [23,48,49], colorectal cancer screening remains particularly vulnerable in many healthcare systems [22,23,34,48]. Possible explanations for the low adherence to FOBT, compared to the other cancer screening tests in Spain, include its later implementation, substantial territorial variability, lower awareness, and difficulties in invitation systems [22,34,48]. Furthermore, the lower adherence could also be explained by specific barriers in the test and the care circuit. Unlike mammography or cytology, FOBT requires the person to complete part of the process at home—collection of the sample and return of the kit—which can reduce its acceptability and make it more necessary to have clear instructions, reminders and reinforcement from primary care. This interpretation is consistent with our results and with Spanish and European studies that show a still low and socially unequal participation in colorectal screening [22,23,34,48].

An unexpected finding was that having better self-rated health was associated with lower FOBT. The reason for this association may be that women who feel healthy have a lower perceived risk and tend to underestimate their personal cancer risk, and therefore believe screening is unnecessary because they have no symptoms [22,23,34,48,49]. Also, women with good health see doctors less often, creating fewer opportunities for physicians to recommend or arrange screening [50]. Our results agree with Onyenemezu et al. who analyzed 177,889 individuals aged 45–75 years interviewed in the 2022 Behavioral Risk Factor Surveillance System finding that those with excellent general health perception were 25% less likely (adjusted odds ratio (AOR) = 0.75; 95% CI = 0.65, 0.88; *p* < 0.0001) to be screened for colorectal cancer compared to those with poor general health perception [50].

Beyond these differences between screening programs, our findings point to the existence of a relatively consistent profile of screening participation. Taken together, the observed associations suggest the presence of an accumulated preventive advantage, characterized by greater exposure to healthcare services. Women who completed all eligible cancer screening tests may represent a subgroup characterized by more frequent interactions with healthcare services, greater engagement with preventive healthcare, higher health awareness, improved access to informational and relational resources, and a greater ability to translate healthcare recommendations into sustained preventive behaviors, rather than simply women responding independently to three separate screening invitations. This interpretation is consistent with previous research showing that the use of preventive services and adherence to health recommendations tend to cluster within specific patient profiles [24,25,26,51,52].

In this context, social support deserves particular attention. In our study, social support did not emerge as a peripheral factor but rather as a consistent marker of adherence across all three screening programs. This finding suggests that participation in cancer screening depends not only on the formal availability of screening services but also on the degree of social integration and relational support available to women. Recent studies have shown that higher levels of perceived social support and living with a partner are associated with greater participation in cancer screening, whereas the accumulation of social vulnerabilities, including lack of social support, is linked to lower adherence to preventive recommendations [25,27,28]. Among immigrant populations in Spain, participation in cervical cancer screening has been shown to depend partly on family networks, partner support, and community integration [29].

Several complementary mechanisms may explain these associations. Social support may facilitate navigation of the healthcare system, improve understanding of preventive recommendations, and increase the confidence required to act upon them [30]. It may also enhance motivation through interpersonal encouragement and social validation while reducing emotional and practical barriers, including fear of screening results, procrastination, and logistical difficulties associated with arranging healthcare appointments [25,28,30]. However, future investigations should clarify which of the many components of social support, such as social integration, family encouragement, healthcare navigation support, or broader socioeconomic advantage, among others, are the ones that really influence women’s adherence to preventive practices.

Consequently, interpreting low screening uptake solely as a consequence of insufficient information or limited healthcare provision may be overly simplistic. Our findings suggest that social support may function simultaneously as a marker of preventive vulnerability and as a potential target for intervention. This interpretation is particularly relevant in the context of a universal healthcare system, where the formal availability of services does not necessarily guarantee their equitable utilization.

Our results also indicate that healthcare utilization and preventive behaviors tend to cluster. Women who had recently consulted primary care or specialist physicians, possessed complementary private health insurance, engaged in regular physical activity, and were adherent to one screening test were also more likely to participate in the remaining screening programs. Cancer screening, therefore, appears to reflect a broader pattern of self-care and engagement with healthcare services rather than isolated preventive actions [24,30,53,54,55,56]. This clustering of preventive behaviors is further supported by the strong associations observed between the different screening programs and by the finding that only 24.4% of eligible women were simultaneously adherent to breast, cervical, and colorectal cancer screening recommendations.

This observation suggests that adequate coverage within individual screening programs does not necessarily translate into comprehensive preventive care. It also points to missed preventive opportunities and a degree of fragmentation in healthcare delivery, whereby contacts with the healthcare system are not consistently used to review all pending preventive interventions [24,53]. Consequently, our findings support the implementation of integrated screening strategies, including coordinated invitations, systematic screening reviews during healthcare encounters, and patient navigation programs, with the aim of reducing inequalities, maximizing the benefits of clinical contacts, and improving overall screening coverage [24,30,54,55,56].

The positive association between physical activity and adherence to all three screening programs can be interpreted within this same framework. Physical activity may reflect a broader orientation toward self-care and a greater capacity to maintain preventive behavior over time, consistent with the well-described healthy-user effect [26,57]. It may also reflect lower levels of social vulnerability and a more stable relationship with healthcare services. This interpretation should not be understood as placing responsibility on women with lower participation rates but rather as highlighting the interconnected nature of lifestyle factors, social position, social networks, and healthcare utilization within a broader preventive continuum.

The inequalities observed according to country of birth further support this broader interpretation. Being born in Spain was independently associated with greater participation in all three screening programs, identifying immigrant status as a major axis of preventive inequality. Both Spanish and international studies consistently report lower participation among immigrant women in breast, cervical, and colorectal cancer screening as a consequence of accumulated administrative, linguistic, occupational, cultural, and health literacy barriers [23,29,47,58,59,60,61]. In this context, immigrant status reflects not only inequalities in access but also lower levels of practical integration into preventive healthcare pathways. From a public health perspective, equity should be considered a central dimension of healthcare quality, consistent with the principles of the Quintuple Aim and with a vision of prevention aimed at reducing inequalities in access to and uptake of effective interventions, particularly among populations at greater risk of exclusion [62,63].

Finally, unlike previous studies, in our investigation, mental health was not associated with adherence to any of the screening tests after multivariable adjustment [64]. A 2025 systematic review concluded that people with mental illnesses participate in organized cancer screening less often than the general population, particularly for breast and cervical cancer [64]. A possible explanation for this lack of association is that in our study, mental health conditions were self-reported, and that patients with more severe mental illness may be less likely to participate in population surveys. Furthermore, Dahò M et al. have reported that psychiatric and neurological conditions have also been recognized as potential sources of healthcare disparities, affecting access to cancer care and eligibility for clinical trials [65]. So future studies should analyze the presence of psychiatric and neurological conditions using clinical reports to assess their real impact on adherence to cancer screening.

From a public health perspective, our findings suggest that the interventions with the greatest potential for impact will be those that are able to simultaneously address program organization, primary care involvement, and social vulnerability. In practical terms, contacts with the health system could be used more systematically to review pending screenings, especially colorectal screening. In addition, reminder systems and patient navigation programs could help reduce barriers to access and facilitate the participation of women with less social support or greater vulnerability. Likewise, educational campaigns should be expanded and improved, both in health centers and among the general population, using classic (radio, television) and modern (internet) information media, pointing out the importance and effectiveness of cancer screening programs. These campaigns should focus especially on the most socially vulnerable groups.

Beyond their immediate public health implications, our findings also identify several priorities for future research. Under one-fourth of women eligible for all three programs were adherent to all three screening tests, and this unacceptably low proportion should be considered when designing public health interventions to improve simultaneous screening. This finding suggests that future studies should move beyond evaluating individual screening programs and instead investigate determinants of comprehensive preventive care. In particular, psychosocial and behavioral factors such as health literacy, perceived cancer risk, physician recommendation, trust in the healthcare system, and mental health deserve further investigation, as these variables may help explain why some women consistently engage in preventive healthcare whereas others remain under-screened despite universal access to healthcare. Longitudinal studies using linked healthcare records would also help clarify the causal pathways underlying adherence to multiple cancer screening programs and support the development of more targeted and equitable prevention strategies.

### Strengths and Limitations

Several strengths of this study should be acknowledged. These include its large sample size, national representativeness, and the simultaneous evaluation of the three cancer screening programs targeting women within the same population. Furthermore, the study incorporated determinants that have received relatively limited attention in previous research, particularly social support and relational integration. However, several limitations should also be considered. First, due to the cross-sectional design, we cannot determine causality or directionality. Second, screening participation was based on self-reported information and is therefore subject to social desirability and recall bias, particularly regarding the timing of the most recent screening test. In addition, the survey does not distinguish whether mammography, cervical cytology, or fecal occult blood testing was performed as part of an organized population-based screening program, through opportunistic screening, or for diagnostic or follow-up purposes. This limitation is particularly relevant for mammography and cervical cytology, which may be performed in symptomatic women, during clinical follow-up, or in the private healthcare sector. Consequently, some misclassification of screening participation is possible, and the reported estimates should be interpreted as the uptake of screening tests rather than exclusive participation in organized screening programs. Third, detailed information regarding formal screening invitations and regional differences in program organization was unavailable. Fourth, the participation rate in the SNHS 2023 reached 62.4%, so a non-response bias must be considered [66]. Fourth, our study did not directly assess structural barriers such as financial constraints, transportation difficulties, and differential access to healthcare services that may substantially influence participation in screening programs, as this information is not collected by the SNHS2023. However, the associations observed with country of birth, health utilization, and social support suggest possible inequalities in the ability to access and complete preventive circuits. This interpretation is consistent with recent studies that link social risks, lower social support, and structural barriers such as geographic inequalities (e.g., rural or underserved areas), financial constraints, transportation difficulties, and differential access to healthcare services, which may substantially influence participation in screening programs. territorial or organizational differences with lower participation in cancer screening programs [22]. These aspects should be considered when designing strategies aimed at socially vulnerable groups [22]. Finally, the adherence intervals and age groups used in this study do not fully coincide with the current screening recommendation. For mammography, we used a 36-month interval for mammography, instead of the 24-month interval recommended by current guidelines, and this may have resulted in slightly higher absolute estimates of adherence by classifying women screened between 24 and 36 months before the interview as adherent. However, as commented before, this approach was adopted to maintain comparability with previous Spanish studies using the same survey methodology and to reduce potential misclassification arising from recall imprecision in self-reported screening dates. Remarkably, for mammography adherence, results of the sensitivity analysis, restricted to women aged 50–69 years, corresponding to the age group included in the organized screening program, produced virtually identical results, supporting the robustness of our findings irrespective of the age range considered.

Nevertheless, these limitations do not undermine the consistency of the patterns observed.

## 5. Conclusions

Adherence to breast, cervical, and especially colorectal cancer screening among Spanish women is below desirable levels and seems stable over time. Participation should not be understood solely as access to screening tests but rather as the expression of a broader preventive health behavior pattern shaped by engagement with healthcare services, social integration, and, particularly, social support. Strategies aimed at improving screening uptake should therefore combine organizational interventions with actions addressing social inequalities and barriers to preventive healthcare participation.

## Figures and Tables

**Table 1:** Adherence to mammography, cervical cytology and fecal occult blood test according to socio-demographic variables among women interviewed in the Spanish National Health Survey 2023.

		MAMMOGRAPHY		CERVICAL CYTOLOGY		FOBT	
Variable	Categories	n (%)	*p* *	n (%)	*p* *	n (%)	*p* *
Age groups for mammography/cervical cytology/FOBT	40 to 45 years	474 (44.2)	<0.001	NA	<0.001	NA	<0.001
	46 to 51/25 to 34/50–54 years	762 (65.3)		643 (60.6)		262 (28.3)	
	52 to 57/35 to 44/55–59 years	924 (84.8)		1133 (68.8)		397 (41.9)	
	58 to 63/45 to 54/60–64 years	948 (81.9)		1285 (67.8)		372 (38.5)	
	64 to 69/55 to 65/65–69 years	889 (78.2)		1256 (59.0)		357 (38.1)	
Country of birth: Spain	No	435 (56.0)	0.001	694 (56.6)	0.007	112 (27.8)	<0.001
	Yes	3562 (73.5)		3623 (65.8)		1276 (37.8)	
Living with a partner	No	1752 (70.1)	0.044	1839 (61.3)	<0.001	600 (33.3)	<0.001
	Yes	2187 (72.5)		2373 (66.8)		774 (40.1)	
Educational level	Ended before 13 years of age:	494 (70.0)	0.103	291 (51.3)	0.01	203 (33.2)	<0.001
	Ended from 13 to 15 years:	935 (71.5)		868 (58.3)		343 (35.0)	
	Ended from 16 to 18 or vocational training	1293 (69.8)		1511 (64.7)		438 (39.0)	
	Completed university studies	1168 (73.4)		1601 (72.7)		369 (40.2)	
Employment status	Working	2254 (70.9)	<0.001	3079 (68.3)	0.664	632 (35.9)	0.664
	Unemployed	464 (65.4)		563 (57.2)		170 (37.0)	
	Retired or disabled	887 (76.8)		342 (57.4)		420 (38.2)	
	Housework or studying	356 (67.9)		290 (50.2)		157 (36.4)	
Household monthly income	Less than 1650 euros	949 (70.9)	0.783	1004 (63.0)	0.19	310 (34.2)	0.134
	From 1650 to less than 2300 euros	614 (72.9)		650 (64.2)		208 (35.8)	
	From 2300 to less than 3800 euros	1163 (71.6)		1251 (64.2)		428 (39.1)	
	From 3800 euros onwards.	1101 (71.8)		1210 (66.4)		384 (37.6)	
Social Support	Poor	215 (62.7)	<0.001	249 (56.7)	<0.001	76 (34.2)	0.002
	Moderate	1411 (68.3)		1535 (62.0)		470 (33.8)	
	Strong	2249 (74.5)		2405 (67.7)		800 (39.4)	
Care giver	No	3239 (69.8)	<0.001	3622 (63.5)	<0.001	1089 (35.8)	0.005
	Yes	731 (79.0)		677 (70.7)		288 (41.4)	

FOBT: fecal occult blood test. NA Not applicable. * *p* value for the association of the screening test with the study variable. NA not applicable.

**Table 2:** Adherence to mammography according to clinical and lifestyle variables among women interviewed in the Spanish National Health Survey 2023.

Variable	Categories	n (%)	*p* Value
Primary care doctor visit in last 12 months	No	453 (55.2)	<0.001
	Yes	3544 (73.8)	
Specialist doctor visit in last 12 months	No	1119 (60.5)	<0.001
	Yes	2878 (76.2)	
ER visit in last 12 months	No	3013 (69.9)	<0.001
	Yes	984 (74.9)	
Private health care insurance coverage	No	3340 (69.3)	<0.001
	Yes	648 (82.4)	
Self-rated health	Fair, bad, very bad	1441 (73.6)	0.002
	Very good, good	2556 (69.7)	
Any chronic condition	No	1097 (63.3)	<0.001
	Yes	2849 (74.9)	
Heart diseases and stroke	No	3760 (70.6)	<0.001
	Yes	237 (80.3)	
Musculoskeletal diseases	No	2275 (65.9)	<0.001
	Yes	1722 (79.3)	
Respiratory disease	No	3597 (70.8)	0.264
	Yes	400 (73.1)	
Gastrointestinal disease	No	3216 (69.4)	<0.001
	Yes	781 (79.0)	
Neurological disease	No	3329 (70.3)	0.002
	Yes	668 (75.4)	
Mental disorders	No	3124 (70.3)	0.010
	Yes	873 (74.1)	
Cancer	No	3727 (70.2)	<0.001
	Yes	270 (86.0)	
Alcohol use	Daily or weekly	1255 (78.8)	<0.001
	Few times a year	1440 (70.0)	
	Not last year or ever	1278 (67.0)	
Tobacco use	Currently	784 (67.3)	<0.001
	Ex smoker	1121 (78.0)	
	Never smoked	2081 (69.5)	
Physically active	No	2860 (68.9)	<0.001
	Yes	1106 (78.4)	
BMI	Normal	1893 (72.2)	0.914
	Overweight	1351 (71.8)	
	Obesity	614 (71.5)	

*p* value for the association of mammography adherence with the study variable. ER: Emergency room; BMI: Body mass index.

**Table 3:** Results of the multivariable logistic regression analysis to identify variables independently associated with adherence to mammography among women interviewed in the Spanish National Health Survey 2023.

Variable	Categories	OR	95% CI
Age groups	40 to 45 years	REFERENCE	-
	46 to 51 years	1.78	1.49–2.13
	52 to 57 years	5.49	4.41–6.82
	58 to 63 years	4.27	3.47–5.26
	64 to 69 years	3.12	2.54–3.82
Country of birth: Spain	No	REFERENCE	-
	Yes	1.32	1.14–1.54
Living with a partner	No	REFERENCE	-
	Yes	1.24	1.08–1.41
Social support	Poor	REFERENCE	-
	Moderate	1.35	1.10–1.69
	Strong	1.72	1.35–2.20
Care giver	No	REFERENCE	-
	Yes	1.14	0.99–1.30
Primary care doctor visit in last 12 months	No	REFERENCE	-
	Yes	1.20	1.02–1.41
Specialist doctor visit in last 12 months	No	REFERENCE	-
	Yes	1.50	1.29–1.73
ER visit in last 12 months	No	REFERENCE	-
	Yes	1.02	0.90–1.17
Private health care insurance coverage	No	REFERENCE	-
	Yes	1.85	1.49–2.29
Musculoskeletal diseases	No	REFERENCE	-
	Yes	1.33	1.15–1.54
Gastrointestinal disease	No	REFERENCE	-
	Yes	1.12	0.98–1.29
Neurological disease	No	REFERENCE	-
	Yes	1.01	0.86–1.19
Mental disorders	No	REFERENCE	-
	Yes	1.02	0.87–1.21
Cancer	No	REFERENCE	-
	Yes	1.70	1.20–2.41
Physically active	No	REFERENCE	-
	Yes	1.68	1.43–1.97

OR: Odds ratios; CI: Confidence interval. ORs were calculated with multivariable logistic regression models. All variables included in the final model are shown in the table.

**Table 4:** Adherence to cervical cytology according to clinical and lifestyle variables among women interviewed in the Spanish National Health Survey 2023.

Variable	Categories	n (%)	*p* Value
Primary care doctor visit in last 12 months	No	612 (52.6)	<0.001
	Yes	3705 (66.5)	
Specialist doctor visit in last 12 months	No	1285 (53.2)	<0.001
	Yes	3032 (70.3)	
ER visit in last 12 months	No	3130 (61.9)	<0.001
	Yes	1187 (71.0)	
Private health care insurance coverage	No	3510 (61.3)	<0.001
	Yes	802 (82.1)	
Self-rated health	Fair, bad, very bad	1285 (64.3)	0.815
	Very good, good	3032 (64.0)	
Any chronic condition	No	1569 (60.1)	<0.001
	Yes	2693 (67.2)	
Heart diseases and stroke	No	4142 (64.0)	0.207
	Yes	175 (67.8)	
Musculoskeletal diseases	No	2942 (62.3)	<0.001
	Yes	1375 (68.3)	
Respiratory disease	No	3874 (63.5)	<0.001
	Yes	443 (70.7)	
Gastrointestinal disease	No	3593 (62.4)	<0.001
	Yes	724 (74.1)	
Neurological disease	No	3584 (63.1)	<0.001
	Yes	733 (69.7)	
Mental disorders	No	3470 (63.3)	0.002
	Yes	847 (68.0)	
Cancer	No	4115 (63.7)	<0.001
	Yes	202 (74.0)	
Alcohol use	Daily or weekly	1321 (71.9)	<0.001
	Few times a year	1714 (65.7)	
	Not last year or ever	1251 (57.1)	
Tobacco use	Currently	922 (63.9)	<0.001
	Ex smoker	1043 (70.3)	
	Never smoked	2341 (62.1)	
Physically active	No	2901 (60.7)	<0.001
	Yes	1386 (74.6)	
BMI	Normal	2319 (67.3)	<0.001
	Overweight	1296 (62.5)	
	Obesity	569 (62.1)	

*p* value for the association of cervical cytology adherence with the study variable. ER: Emergency room; BMI: Body mass index.

**Table 5:** Results of the multivariable logistic regression analysis to identify variables independently associated with adherence to cervical cytology among women interviewed in the Spanish National Health Survey 2023.

Variable	Categories	OR	95% CI
Age groups	25 to 34 years	REFERENCE	-
	35 to 44 years	1.25	1.06–1.47
	45 to 54 years	1.17	0.99–1.38
	55 to 65 years	0.92	0.78–1.09
Country of birth: Spain	No	REFERENCE	-
	Yes	1.19	1.03–1.37
Living with a partner	No	REFERENCE	-
	Yes	1.21	1.08–1.35
Educational level	Ended before 13 years of age:	REFERENCE	-
	Ended from 13 to 15 years:	0.97	0.81–1.16
	Ended from 16 to 18 or vocational training	1.23	1.04–1.46
	Completed university studies	1.64	1.37–1.97
Social support	Poor	REFERENCE	-
	Moderate	1.26	1.02–1.55
	Strong	1.58	1.29–1.94
Care giver	No	REFERENCE	-
	Yes	1.15	0.95–1.41
Primary care doctor visit in last 12 months	No	REFERENCE	-
	Yes	1.23	1.06–1.43
Specialist doctor visit in last 12 months	No	REFERENCE	-
	Yes	1.62	1.43–1.84
ER visit in last 12 months	No	REFERENCE	-
	Yes	1.10	0.95–1.27
Private health care insurance coverage	No	REFERENCE	-
	Yes	2.20	1.83–2.65
Musculoskeletal diseases	No	REFERENCE	-
	Yes	1.21	1.05–1.39
Gastrointestinal disease	No	REFERENCE	-
	Yes	1.03	0.87–1.19
Cancer	No	REFERENCE	-
	Yes	1.47	1.08–1.99
Physically active	No	REFERENCE	-
	Yes	1.48	1.29–1.68
BMI	Normal	REFERENCE	-
	Overweight	0.87	0.77–0.99
	Obesity	0.85	0.72–0.99

OR: Odds ratios. CI: Confidence interval. ORs were calculated with multivariable logistic regression models. All variables included in the final model are shown in the table. BMI: Body mass index.

**Table 6:** Adherence to the fecal occult blood test according to clinical and lifestyle variables among women interviewed in the Spanish National Health Survey 2023.

Variable	Categories	n (%)	*p* Value
Primary care doctor visit in last 12 months	No	90 (18.7)	<0.001
	Yes	1298 (39.4)	
Specialist doctor visit in last 12 months	No	304 (25.6)	<0.001
	Yes	1084 (41.9)	
ER visit in last 12 months	No	989 (34.0)	<0.001
	Yes	399 (45.8)	
Private health care insurance coverage	No	1172 (35.7)	<0.001
	Yes	214 (44.6)	
Self-rated health	Fair, bad, very bad	621 (43.0)	<0.001
	Very good, good	767 (32.9)	
Any chronic condition	No	249 (25.7)	<0.001
	Yes	1125 (40.8)	
Heart diseases and stroke	No	1268 (36.0)	<0.001
	Yes	120 (48.0)	
Musculoskeletal diseases	No	630 (30.9)	<0.001
	Yes	758 (43.7)	
Respiratory disease	No	1238 (36.5)	0.303
	Yes	150 (39.2)	
Gastrointestinal disease	No	1043 (34.2)	<0.001
	Yes	345 (47.4)	
Neurological disease	No	1139 (35.6)	<0.001
	Yes	249 (43.5)	
Mental disorders	No	1032 (34.9)	<0.001
	Yes	356 (43.7)	
Cancer	No	1254 (35.7)	<0.001
	Yes	134 (51.0)	
Alcohol use	Daily or weekly	443 (42.1)	<0.001
	Few times a year	449 (34.1)	
	Not last year or ever	485 (35.5)	
Tobacco use	Currently	245 (32.4)	<0.001
	Ex smoker	464 (44.2)	
	Never smoked	675 (34.6)	
Physically active	No	987 (34.4)	<0.001
	Yes	391 (45.3)	
BMI	Normal	632 (38.7)	0.045
	Overweight	468 (34.6)	
	Obesity	238 (38.7)	

*p* value for the association of fecal occult blood test adherence with the study variable. ER: Emergency room. BMI: Body mass index.

**Table 7:** Results of the multivariable logistic regression analysis to identify variables independently associated with adherence to the fecal occult blood test among women interviewed in the Spanish National Health Survey 2023.

Variable	Categories	OR	95% CI
Age groups	50–54 years	REFERENCE	
	55–59 years	1.69	1.38–2.08
	60–64 years	1.55	1.26–1.91
	65–69 years	1.45	1.17–1.78
Country of birth: Spain	No	REFERENCE	-
	Yes	1.47	1.20–1.87
Living with a partner	No	REFERENCE	-
	Yes	1.36	1.18–1.56
Educational level	Ended before 13 years of age:	REFERENCE	-
	Ended from 13 to 15 years:	1.15	0.92–1.43
	Ended from 16 to 18 or vocational training	1.44	1.16–1.79
	Completed university studies	1.55	1.24–1.94
Social support	Poor	REFERENCE	-
	Moderate	1.04	0.92–1.17
	Strong	1.31	1.02–1.63
Primary care doctor visit in last 12 months	No	REFERENCE	-
	Yes	2.10	1.61–2.74
Specialist doctor visit in last 12 months	No	REFERENCE	-
	Yes	1.48	1.24–1.75
ER visit in last 12 months	No	REFERENCE	-
	Yes	1.172	0.97–1.42
Private health care insurance coverage	No	REFERENCE	-
	Yes	1.27	1.03–1.57
Self-rated health	Fair, bad, very bad	REFERENCE	-
	Very good, good	0.84	0.72–0.97
Musculoskeletal diseases	No	REFERENCE	-
	Yes	1.27	1.09–1.49
Gastrointestinal disease	No	REFERENCE	-
	Yes	1.24	1.03–1.49
Mental disorders	No	REFERENCE	-
	Yes	1.19	097–1.47
Cancer	No	REFERENCE	-
	Yes	1.36	1.04–1.79
Physically active	No	REFERENCE	-
	Yes	1.52	1.28–1.80

OR: Odds ratios. CI: Confidence interval. ORs were calculated with multivariable logistic regression models. All variables included in the final model are shown in the table.

**Table 8:** Odds ratios for adherence to two cancer screening tests simultaneously among women interviewed in the Spanish National Health Survey 2023.

Screening Tests	Odds Ratio	95% Confidence Interval
Mammography & cervical cytology	4.37	3.84–4.98
Mammography & fecal occult blood test	3.88	3.14–4.81
Cervical cytology & fecal occult blood test	1.69	1.45–1.97

## Data Availability

Data from the Spanish National Health Survey 2023 can be freely downloaded from https://www.sanidad.gob.es/estadisticas/microdatos.do (accessed on 12 June 2026).

## Supplementary Materials

The following supporting information can be downloaded at https://www.mdpi.com/article/10.3390/healthcare14142175/s1, Table S1: Definition of variables according to the questions included in the Spanish National Health Survey 2023; Table S2. Distribution of the study population (women aged 25 to 69 years) according to socio-demographic, clinical and lifestyle variables. Spanish National Health Survey 2023. Table S3. Adherence to mammography according to socio-demographic, clinical, and lifestyle variables among women aged 50 to 69 years interviewed in the Spanish National Health Survey 2023. Table S4. Results of the multivariable logistic regression analysis to identify variables independently associated with adherence to mammography among women aged 50 to 69 years interviewed in the Spanish National Health Survey 2023.

## Abbreviations

The following abbreviations are used in this manuscript:

EsdE2023	Encuesta de Salud de España, (2023 Spanish Health Survey)
FOBT	Fecal occult blood testing
OR	Odds ratio
CI	Confidence interval
