# Supplementary material for: Adherence to Breast, Cervical, and Colorectal Cancer Screening Among Women in Spain: Evidence from the 2023 Spanish Health Survey
Source: Healthcare (Basel). 2026 Jul 18;14(14):2175. doi: 10.3390/healthcare14142175 (PMC13411259; doi:10.3390/healthcare14142175)
Supplement: Supplementary file 1 [file healthcare-14-02175-s001.zip › healthcare-4409509-supplementary.pdf]

Table S1. Definition of variables according to the questions included in the Spanish National Health Survey 2023.

Questions	Description and answer	Variables name	Categories
Which is your sex? Selected only women	1. Men<br>2. Women	Sex	Selected only women
Age in years. Selected those aged 25 to 69 years	Age groups		Defined according to screening test
Uptake of breast cancer screening. It was determined by asking: 'Have you ever undergone a mammogram?'. Those who answered affirmatively were asked a second question, "When was the last time you had a mammogram?"	1. In the last 12 months<br>2. Over 1 year but less than 2 years ago<br>3. Over 2 years but less than 3 years ago<br>4. Over 3 years ago	Mammogram	1. Yes: Options 1 to 3<br>2. No: Option 4
Uptake of cervical cancer screening. It was determined by asking:<br>'Have you ever undergone cervical cytology?'.<br>Women who answered affirmatively were asked,<br>'When was the last time you underwent cervical cytology?	1. In the last 12 months<br>2. Over 1 year but less than 2 years ago<br>3. Over 2 years but less than 3 years ago<br>4. Over 3 years ago	Cervical cytology	1. Yes: Options 1 to 3<br>2. No: Option 4
Uptake of colorectal cancer screening. It was determined by asking:<br>'Have you ever undergone a fecal occult blood test?'. Those who answered affirmatively were asked a second question, 'When was the last time you had a fecal occult blood test?	1. In the past 12 months<br>2. Over 1 year but less than 2 years ago<br>3. Over 2 years but less than 3 years ago<br>4. Over 3 years but less than 5 years ago<br>5. Over 5 years ago	FOBT	1. Yes: Options 1 and 2<br>2. No: Options 3 to 5
What is your country of birth?	1. Spain<br>2. Foreign	Country of birth	1. Spain<br>2. Abroad
Of the following options, which describes your current situation?	1. Living with your spouse<br>2. Living with a common-law partner<br>3. Not living together as a couple	Living with a partner	1. Yes: Option 1 and 2<br>2. No: option 3
What level of education have you completed?	1. Does not know how to read or write<br>2. Incomplete primary education<br>3. Complete primary education<br>4. First stage of Secondary Education, with or without a qualification<br>5. Elementary Spanish Upper Secondary Education<br>6. Upper secondary education<br>7. Intermediate vocational training or equivalent	Educational level	1. Ended before 13 years of age: Options 1 to 3<br>2. Ended from 13 to 15 years: Options 4 and 5<br>3. Ended from 16 to 18 or vocational training. Options 6 to 8.<br>4 Completed university studies: Options 9 and 10

	8. Advanced vocational training or equivalent<br>9. University studies or equivalent<br>10. University (master, PhD….)		
Which is your employment status?	1. Working<br>2. Unemployed<br>3. Retired<br>4. Studying<br>5. Unable to work<br>6. Housework<br>7. Other	Employment status	1. Working. Option 1<br>2. Unemployed. Option 2<br>3. Retired, or disabled. Options 3 and 5<br>4. Housework or studying. Options 4 and 6
If you don't know the exact value of household income, could you tell us which of the following ranges best represents your entire household's net monthly income, after deductions for taxes, Social Security, etc.?	1. Less than 1100 euros<br>2. From 1100 to less than 1650 euros<br>3. From 1650 to less than 2300 euros<br>4. From 2300 to less than 3800euros<br>5. From 3800 euros onwards	Household monthly income	1. Less than 1650 euros. Options 1 and 2<br>2. From 1650 to less than 2300 euros. Option 3<br>3. .From 2300 to less than 3800 euros. Option 4<br>4. From 3800 euros onwards. Option 5
**The Oslo Social Support Scale. Question 1**<br>In case you have a serious personal problem of any kind, how many people close to you could you count on?	1. None<br>2. 1 or 2 people<br>3. From 3 to 5 people<br>4. More than 5 people	Social support	The sum score ranges from 3 to 14, with high values representing strong levels and low values representing poor levels of social support.<br>We defined three categories*<br>1. 3–8 poor social support.<br>2. 9–11 moderate social support.<br>3. 12–14 strong social support.
**The Oslo Social Support Scale. Question 2**<br>To what extent are other people interested in what happens to you? We are referring to both family members and people who are not members of your family.	1. None<br>2. Little<br>3. Not much<br>4. Some<br>5. A lot		
**The Oslo Social Support Scale. Question 3**<br>How easy it is to get help from neighbors in case of need	1. Very difficult<br>2. Difficult<br>3. It is possible<br>4. Easy<br>5. Very easy		
Do you take care of an elderly person or someone who has a chronic ailment at least once a week? Don't consider it if it's part of your job.	1. Yes<br>2. No	Care giver	1. Yes<br>2. No

When was the last time you consulted your primary care doctor?	1. In the last 4 weeks<br>2. Between 4 weeks and 12 months .<br>3. 12 months or more ago<br>4. Never	Primary care doctor visit in last 12 months	1. Yes. Options 1 and 2.<br>2. No. Options 3 and 4
When was the last time you consulted a specialist doctor?	1. In the last 4 weeks<br>2. Between 4 weeks and 12 months .<br>3. 12 months or more ago<br>4. Never	Specialist doctor visit in last 12 months	1. Yes. Options 1 and 2.<br>2. No. Options 3 and 4
In the last 12 months, have you visited the emergency room?	1. Yes<br>2. No	Emergency room visit in last 12 months	1. Yes<br>2. No
What type of health insurance coverage do you have?	Respondents were classified according to their type of health insurance coverage	Private health insurance coverage	1. Yes<br>2. No
In the past twelve months, how is your perception of your general health status?	1. Very good<br>2. Good<br>3. Fair<br>4. Bad<br>5. Very bad	Self-rated health	1. Very good/good: Options 1 and 2<br>2. Fair/poor/very poor: Options 3 to 5
Do you have any chronic or long-term health problems or diseases? We understand for a long duration if the health problem or disease has lasted or is expected to last 6 months or longer.	1. Yes<br>2. No	Any chronic conditions	1. Yes<br>2. No
"Has your doctor told you that you are suffering from…?"<br>• Myocardial infarction<br>• Angina/coronary.<br>• Heart failure<br>• Other heart diseases<br>• Stroke	Respondents reporting any of the listed cardiovascular conditions were classified as having heart diseases and stroke	Heart diseases and stroke	1. Yes (any item = Yes)<br>2. No (all items = No)
"Has your doctor told you that you are suffering from…?"<br>• Osteoarthritis (excluding arthritis);<br>• Chronic cervical back pain<br>• Chronic lumbar back pain<br>• Osteoporosis	Respondents reporting any of the listed musculoskeletal conditions were classified as having musculoskeletal disorders	Musculoskeletal disease	1. Yes (any item = Yes)<br>2. No (all items = No)

"Has your doctor told you that you are suffering from…?"<br>• Asthma<br>• Chronic bronchitis/emphysema/COPD	Respondents reporting any of the listed respiratory conditions were classified as having respiratory disease	Respiratory diseases	1. Yes (any item = Yes)<br>2. No (all items = No)
"Has your doctor told you that you are suffering from…?"<br>1. Gastric or duodenal ulcer<br>2. Chronic constipation<br>3. Cirrhosis/liver dysfunction	Respondents reporting any of the listed gastrointestinal conditions were classified as having chronic gastrointestinal disorder	Gastrointestinal diseases	1. Yes (any item = Yes)<br>2. No (all items = No)
"Has your doctor told you that you are suffering from…?"<br>• Alzheimer's disease or other dementias.<br>• Migraine or frequent headache	Respondents reporting any of the listed neurological conditions were classified as having neurological disorder	Neurological diseases	1. Yes (any item = Yes)<br>2. No (all items = No)
"Has your doctor told you that you are suffering from…?"<br>• Depression<br>• Anxiety<br>• Other mental health problems	Respondents reporting any of the listed mental health conditions were classified as having mental health disorder	Mental health disorders	1. Yes (any item = Yes)<br>2. No (all items = No)
Has your doctor told you that you are suffering from malignant tumors?	Respondents reporting a history of malignant tumors	Cancer	1. Yes 2. No
During the past 12 months, how often have you had alcoholic beverages of any kind (i.e. beer, wine, spirits, distilled and mixed drinks, or other alcoholic beverages)?	1. Daily or almost daily<br>2. 5-6 days per week<br>3. 3-4 days per week<br>4. 1-2 days per week<br>5. 2-3 days in a month<br>6. Once a month<br>7. Less than once a month<br>8. Not in the last 12 months have I stopped drinking<br>9. Never or just a few sips to taste it throughout life	Alcohol use	1. Daily or weekly Options 1 to 4<br>2. Few times a year Options 5 to 7<br>3 Not last year or ever. Options 8 and 9
Could you tell me if you smoke?	1. Yes, I smoke daily<br>2. Yes, I smoke, but not daily<br>3. I don't currently smoke but have smoked before<br>4. I neither smoke nor have I ever smoked regularly	Tobacco use	1. Currently Options 1 and 2<br>2. Ex-smoker: Options 3<br>3. Never smoked. Option 4
Which of these possibilities best describes how often you do some physical activity in your free time?	1. I don't exercise. I occupy my free time almost completely sedentary.	Physically active	1. No: Option 1<br>2. Yes: Option 2 to 4

	2. I do some occasional physical or sports activities<br>3. I do physical activity several times a month<br>4. I do sports or physical training several times a week		
1. Could you tell me how tall you are, approximately, without shoes?<br>2. Could you tell me your weight, approximately, without shoes and clothes?	Body mass index is calculated with the formula:<br>Weight in kg/ (Height in meters)^2	Body mass index (BMI)	1. Normal (<25)<br>2. Overweight (25-29.9)<br>3. Obesity (≥30)

* Kocalevent RD, Berg L, Beutel ME, Hinz A, Zenger M, Härter M, Nater U, Brähler E. Social support in the general population: standardization of the Oslo social support scale (OSSS-3). BMC Psychol. 2018;6:31. doi: 10.1186/s40359-018-0249-9.

Table S2. Distribution of the study population (women aged 25 to 69 years) according to socio-demographic, clinical and lifestyle variables. Spanish National Health Survey 2023

**Variable**	**Categories**	**n**	**%**
Age groups	25 to 39 years	1830	24.6
	40 to 49 years	1848	24.8
	50 to 59 years	1873	25.1
	60 to 69	1903	25.5
Country of birth	Spain	6183	82.9
Living with a partner	Yes	3918	53.8
Educational level	Ended before 13 years of age:	789	10.9
	Ended from 13 to 15 years:	1642	22.6
	Ended from 16 to 18 or vocational training	2508	34.5
	Completed university studies	2325	32.0
Employment status	Working	4535	61.4
	Unemployed	990	13.4
	Retired or disabled	1179	16.0
	Homework or studying	680	9.2
Household monthly income	Less than 1650 euros	1773	25.1
	From 1650 to less than 2300 euros	1118	15.8
	From 2300 to less than 3800 euros	2163	30.6
	From 3800 euros onwards.	2007	28.4
Social support	Poor	484	6.7
	Moderate	2738	38.2
	Strong	3949	55.1
Care giver	Yes	1032	14.0
Primary care doctor visit in last 12 months	Yes	6223	83.5
Specialist doctor visit in last 12 months	Yes	4813	64.6
ER visit in last 12 months	Yes	1821	24.4
Private health care insurance coverage	Yes	1055	14.2
Self-rated health	Very good, good	5139	68.9
Any chronic condition	Yes	4602	62.7
Heart diseases and stroke	Yes	331	4.4
Musculoskeletal diseases	Yes	2405	32.3
Respiratory diseases	Yes	702	9.4
Gastrointestinal diseases	Yes	1140	15.3
Neurological diseases	Yes	1147	15.4
Mental disorders	Yes	1421	19.1
Cancer	Yes	334	4.50
Alcohol use	Daily or weekly	2013	27.3
	Few times a year	2848	38.7
	Not last year or ever	2500	34.0
Tobacco use	Currently	1548	20.9
	Ex smoker	1682	22.7
	Never smoked	4186	56.4
Physically active	Yes	1995	27.1
BMI	Normal	3720	52.2
	Overweight	2345	32.9
	Obesity	1056	14.8

ER: Emergency room. BMI: Body mass index

Table S3. Adherence to mammography according to socio-demographic, clinical and lifestyle variables among women aged 50 to 69 years interviewed in the Spanish National Health Survey 2023

Variable	Categories	n ()	P*
Age groups	50-54 years	749(81.0)	<0.001
	55-59 years	807 (85.1)	
	60-64 years	765 (79.2)	
	65-69 years	736 (78.5)	
Country of birth Spain	No	282 (70.0)	<0.001
	Yes	2775(82.3)	
Living with a partner	No	1441 (78.3)	<0.001
	Yes	1608 (83.4)	
Educational level	Ended before 13 years of age:	458 (75.0)	<0.001
	Ended from 13 to 15 years:	798 (81.5)	
	Ended from 16 to 18 or vocational training	920 (81.9)	
	Completed university studies	781 (85.2)	
Employment status	Working	1483 (84.3)	<0.001
	Unemployed	366 (79.6)	
	Retired or disable	862 (78.4)	
	Housework or studying	324 (75.2)	
Household monthly income	Less than 1650 euros	722 (79.7)	0.445
	From 1650 to less than 2300 euros	479 (82.4)	
	From 2300 to less than 3800 euros	900 (82.2)	
	From 3800 euros onwards.	834 (81.7)	
Social Support	Poor	670 (75.9)	<0.001
	Moderate	1171 (81.7)	
	Strong	1182 (84.0)	
Care giver	No	2429 (79.7)	<0.001
	Yes	609 (87.5)	
Primary care doctor visit in last 12 months	No	326 (67.8)	<0.001
	Yes	2731 (82.9)	
Specialist doctor visit in last 12 months	No	861 (72.5)	<0.001
	Yes	2196 (84.8)	
ER visit in last 12 months	No	2316 (79.7)	<0.001
	Yes	741 (85.1)	
Private health care insurance coverage	No	2625 (79.9)	<0.001
	Yes	426 (88.8)	
Self-rated health	Fair, bad, very bad	1173 (81.2)	0.736
	Very good, good	1884 (80.8)	
Any chronic condition	No	717 (73.9)	<0.001
	Yes	2307 (83.6)	
Heart diseases and stroke	No	2843 (80.6)	0.053
	Yes	214 (85.6)	
Musculoskeletal diseases	No	1572 (77.0)	<0.001
	Yes	1485 (85.6)	
Respiratory disease	No	2740 (80.8)	0.342
	Yes	317 (82.8)	
Gastrointestinal disease	No	2417 (79.3)	<0.001
	Yes	640 (87.9)	

Table S3(Continued). Adherence to mammography according to socio-demographic, clinical and lifestyle variables among women aged 50 to 69 years interviewed in the Spanish National Health Survey 2023

Neurological disease	No	2565 (80.1)	<0.001
	Yes	492 (85.9)	
Mental disorders	No	2371 (80.1)	0.008
	Yes	686 (84.2)	
Cancer	No	2828 (80.5)	0.009
	Yes	229 (87.1)	
Alcohol use	Daily or weekly	934 (88.8)	<0.001
	Few times a year	1071 (81.3)	
	Not last year or ever	1034 (75.6)	
Tobacco use	Currently	595 (78.7)	<0.001
	Ex smoker	911 (86.8)	
	Never smoked	1544 (79.1)	
Physically active	No	2243 (78.2)	<0.001
	Yes	789 (91.3)	
BMI	Normal	1363 (83.6)	0.913
	Overweight	1085 (80.2)	
	Obesity	507 (82.4)	

* p value for the association of mammography with the study variable. ER: Emergency room. BMI; Body mass index

Table S4. Results of the multivariable logistic regression analysis to identify variables independently associated with adherence to mammography among women aged 50 to 69 years interviewed in the Spanish National Health Survey 2023

**Variable**	**Categories**	**OR**	**95% CI**
Age groups	50-54 years	REFERENCE	-
	55-59 years	1.38	1.03- 1.85
	60-64 years	1.06	0.79-1.41
	65-69 years	0.93	0.74-1.17
Country of birth Spain	No	REFERENCE	-
	Yes	1.48	1.15- 1.89
Living with a partner	No	REFERENCE	
	Yes	1.28	1.07-1.53
Social Support	Poor	REFERENCE	-
	Moderate	1.12	0.81 -1.56
	Strong	1.71	1.23 -2.38
Care giver	No	REFERENCE	-
	Yes	1.22	0.84-1.82
Primary care doctor visit in last 12 months	No	REFERENCE	-
	Yes	1.49	1.18 -1.90
Specialist doctor visit in last 12 months	No	REFERENCE	-
	Yes	1.68	1.34 -1.98
ER visit in last 12 months	No	REFERENCE	-
	Yes	1.06	0.91-1.37
Private health care insurance coverage	No	REFERENCE	-
	Yes	1.49	1.17-1.89
Musculoskeletal diseases	No	REFERENCE	-
	Yes	1.63	1.35-1.97
Neurological disease	No	REFERENCE	-
	Yes	1.13	0.95 -1.34
Mental disorders	No	REFERENCE	-
	Yes	1.09	.81-1.49
Cancer	No	REFERENCE	-
	Yes	1.45	1.05-2.05
Physically active	No	REFERENCE	-
	Yes	2.15	1.14-2.87

OR: Odds ratios. CI: Confidence interval. ORs were calculated with multivariable logistic regression models. All variables included in the final model are shown in the table
